# Improving Prehospital Stroke Services in Rural and Underserved Settings With Mobile Stroke Units

**DOI:** 10.3389/fneur.2019.00159

**Published:** 2019-03-01

**Authors:** Shrey Mathur, Silke Walter, Iris Q. Grunwald, Stefan A. Helwig, Martin Lesmeister, Klaus Fassbender

**Affiliations:** ^1^Department of Neurology, Saarland University Medical Centre, Homburg, Germany; ^2^Neuroscience Unit, Faculty of Medicine, Anglia Ruskin University, Chelmsford, United Kingdom; ^3^Department of Medicine, Southend University Hospital NHS Foundation Trust, Westcliff-on-Sea, United Kingdom

**Keywords:** mobile stroke unit, rural health, prehospital, telemedecine, telestroke

## Abstract

In acute stroke management, time is brain, as narrow therapeutic windows for both intravenous thrombolysis and mechanical thrombectomy depend on expedient and specialized treatment. In rural settings, patients are often far from specialized treatment centers. Concurrently, financial constraints, cutting of services and understaffing of specialists for many rural hospitals have resulted in many patients being underserved. Mobile Stroke Units (MSU) provide a valuable prehospital resource to rural and remote settings where patients may not have easy access to in-hospital stroke care. In addition to standard ambulance equipment, the MSU is equipped with the necessary tools for diagnosis and treatment of acute stroke or similar emergencies at the emergency site. The MSU strategy has proven to be effective at facilitating time-saving stroke triage decisions. The additional on-board imaging helps to determine whether a patient should be taken to a primary stroke center (PSC) for standard treatment or to a comprehensive stroke center (CSC) for advanced stroke treatment (such as intra-arterial therapy) instead. Diagnosis at the emergency site may prevent additional in-hospital delays in workup, handover and secondary (inter-hospital) transport. MSUs may be adapted to local needs—especially in rural and remote settings—with adjustments in staffing, ambulance configuration, and transport models. Further, with advanced imaging and further diagnostic capabilities, MSUs provide a valuable platform for telemedicine (teleradiology and telestroke) in these underserved areas. As MSU programmes continue to be implemented across the world, optimal and adaptable configurations could be explored.

## Introduction

Stroke is one of the most frequent causes of disability and death worldwide (1, 2). Acute ischaemic stroke has enormous societal and financial costs due to rehabilitation, long-term care, and lost productivity (3). Due to rapid advances over the last decades, there are now safe and effective treatments for stroke (4, 5). However, leading therapeutic modalities, intravenous thrombolysis, and mechanical thrombectomy are extremely time-dependent (6–9). International guidelines now support the use of thrombolysis up to 4.5 h after symptom onset as well as mechanical thrombectomy as a viable option within 6–24 h of last known normal for select patients with a mismatch between clinical deficit and infarct (4, 5). However, urgency remains as in case of mechanical thrombectomy, as for each 30 min delay before reperfusion, the relative likelihood of a good clinical outcome decreases by approximately 15% (10, 11).

With this in mind, concerted efforts have been made to improve in-hospital management of stroke by minimizing delays and optimizing protocols and personnel. However, despite substantial efforts to streamline care, a limited number of patients receive thrombolysis and fewer than 1–2% receive mechanical thrombectomy (12, 13). According to a recent survey of European stroke experts, 7.3% of ischaemic stroke patients in Europe received thrombolysis with 13 countries reporting rates higher than 10% with the highest rates in the Netherlands (20.6%), Denmark (19.%) and Austria (18.4%), and lowest of <1% (14). Thrombolysis rates vary both between and within nations, with rates as high as 28.5% in the German state of Baden-Württemberg compared to the national-level rate of 17.5% in Germany (14, 15). Furthermore, thrombolysis rates have continued to increase over time—for example, increasing from 4.0% (2003–2005) to 7.0% (2010–2011) in the United States—and continue to increase after publication of large trials (such as ECASS III) which have expanded 3- to 4.5-h since onset window (13, 16). These low rates of treatment are largely due to the fact that patients do not reach the hospital in time for assessment and treatment within the narrow therapeutic windows. In fact, only 15–60% of acute stroke patients arrive at the hospital within 3 h after onset of symptoms (17, 18).

**Figure 1:**
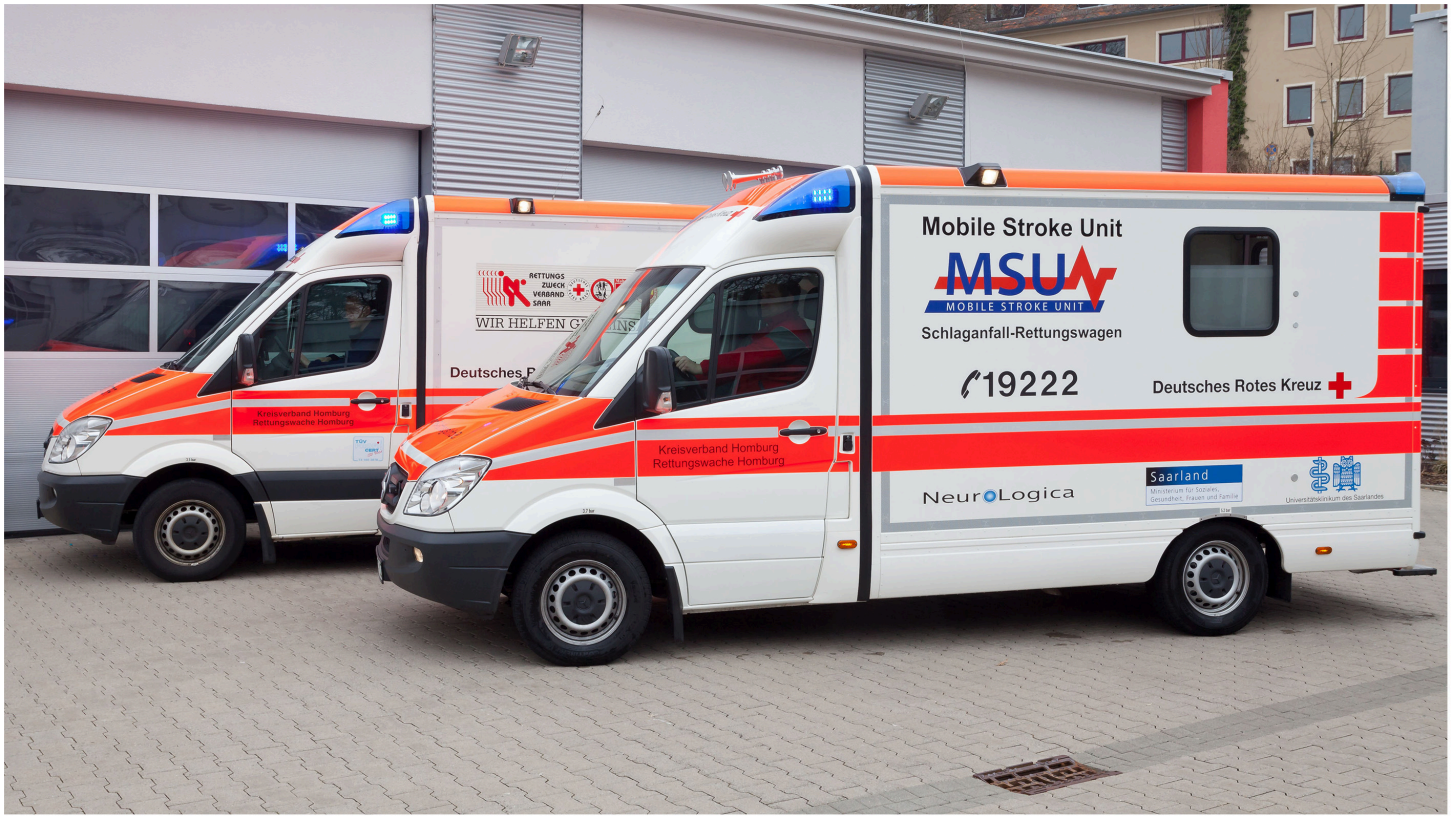
Mobile Stroke Unit (MSU). The Mobile Stroke Unit is an ambulance which contains a multimodal CT scanner, a point-of-care laboratory, as well as a telemedicine system, which allows transfer of CT images and videos of patient examination for input from hospital specialists. Pictured is the MSU in Homburg, Germany.

The Mobile Stroke Unit (MSU)—first proposed and studied in Homburg, Germany—is an innovation in the prehospital phase which aims to address this urgency by bringing the hospital to the patient (Figure 1) (19, 20). This is achieved by equipping an ambulance with the necessary tools for diagnosis and treatment of acute stroke or similar, time-sensitive emergencies. Thus, in addition to standard ambulance equipment, the MSU is equipped with a small-bore portable CT scanner, a point-of-care laboratory, and stroke medication. Via incorporated telemedicine, CT images, and real-time videos can be bidirectionally transmitted between the hospital and ambulance for expert consultation. These images can be integrated with hospital medical records. With the point-of-care laboratory, hematological parameters (thrombocytes, erythrocytes, leukocytes, hemoglobin), coagulation parameters [international normalized ratio (INR), activated partial thromboplastin time (aPTT)], clinical chemistry parameters (gamma-glutamyltransferase, pancreatic amylase, creatinine, glucose), and others can be analyzed within minutes in the MSU (19–36).

With MSUs, earlier thrombolytic therapy—within the first, or golden, hour—after acute ischaemic stroke has been shown to be beneficial for patients with improved functional outcomes—for both patients who were independent and those who needed assistance in activities of daily living before their stroke (28, 37–40). When compared to hospital-based thrombolysis in the first hour after acute stroke, MSUs have been shown to have comparable functional outcomes and mortality at 3 months (41). MSUs have been shown to facilitate shorter alarm-to-treatment times without increasing adverse events (such as secondary intracerebral hemorrhage) (19, 28).

MSUs have been shown to play an integral role in evolving stroke services. They have been demonstrated to be effective in improving key prehospital temporal metrics (such as alarm-to-therapy and alarm-to-imaging times) in many centers worldwide (19, 25, 28, 29, 34, 42, 43). However, there remain relevant stroke treatment gaps for rural patients. MSU models and services can be adapted to improve stroke services for these patients. In addition to acute stroke triage, here, MSUs can provide telemedicine services to patients who are underserved. As stroke services continue to evolve, it is important to consider both the core and additional services which can be provided by MSUs.

## Incorporating MSUs into Stroke Service Planning

### Primary Stroke Centers and Comprehensive Stroke Centers

**Figure 2:**
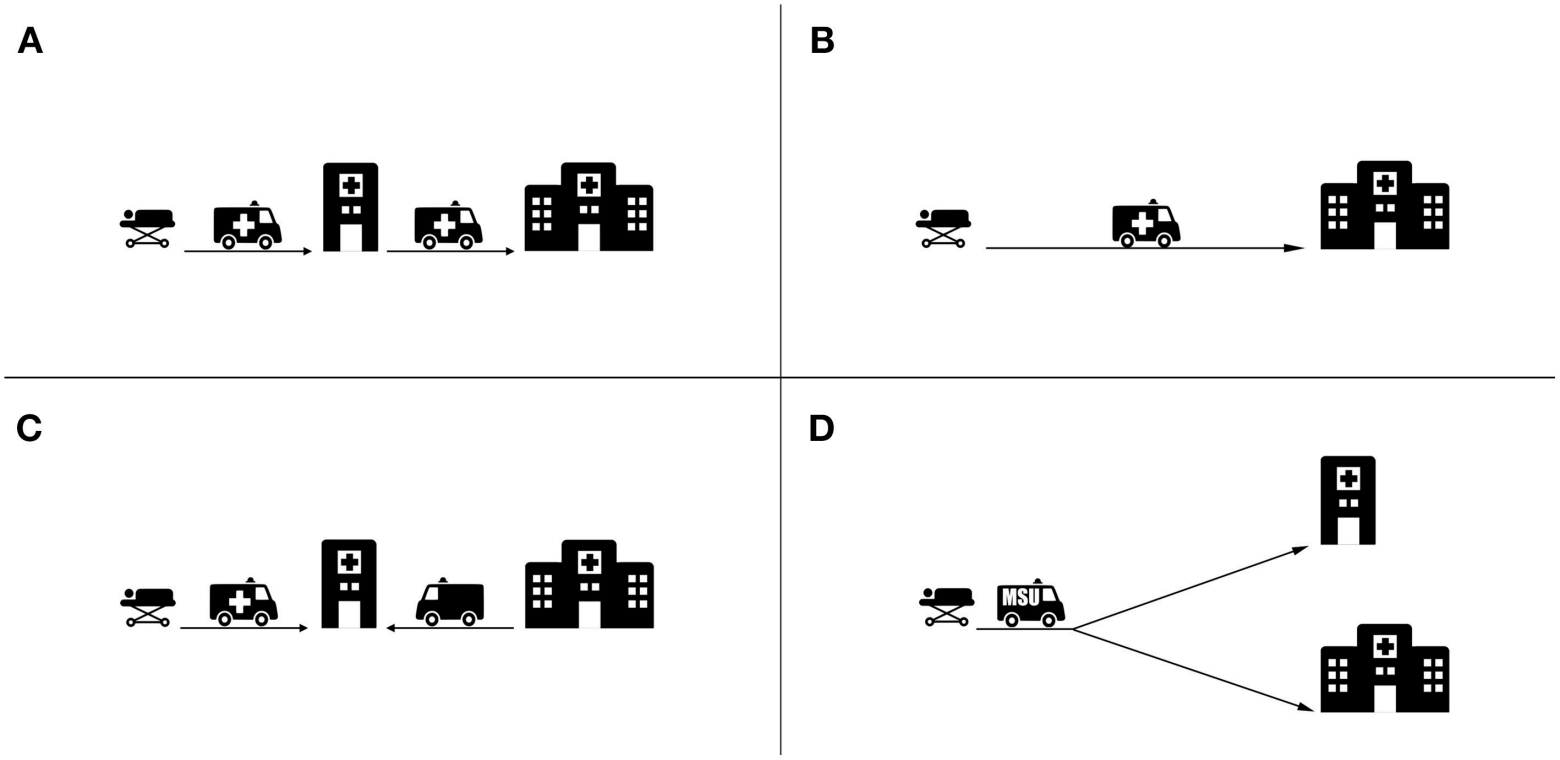
Main transport strategies for acute stroke patients. **(A)** Drip and Ship strategy whereby the patient is transported from the emergency site to a PSC for thrombolysis and then further transported to a CSC for thrombectomy. **(B)** Mothership strategy whereby the patients is transported directly to the CSC, bypassing the PSC. **(C)** Specialist Rendezvous strategy (sometimes called “flying” doctor) whereby the patient arriving at the PSC is met by an interventionalist from a CSC. **(D)** MSU Strategy whereby triage decisions are made at the emergency site and the patient is transported based on the diagnosis to a PSC or CSC where appropriate.

The organization of acute stroke care has evolved significantly during the past few decades (Figure 2) (44). Primary Stroke Centers (PSCs) have been implemented to improve stroke care. PSCs include acute stroke teams, stroke units, written care protocols, and an integrated emergency response system (45). Comprehensive Stroke Centers (CSCs) integrate specialized services for the management of most severe cerebrovascular disease. These are typically staffed with experts in neurointervention and vascular neurology, have advanced round-the-clock, neuroimaging capabilities including MRI and cerebral angiography, specialize in surgical and endovascular techniques (including clipping and coiling of intracranial aneurysms, carotid endarterectomy, and intra-arterial thrombolytic therapy), and have specific infrastructure such as an intensive care unit (46).

As of 2017, comprehensive stroke centers accounted for roughly one-third of all stroke centers in the United States (327 of 1,148) and in France (37 of 132) (47–49). Considering large, densely populated European countries, the number of endovascular therapy capable centers range from 135 in Germany (1.7 per million inhabitants) to 28 in the United Kingdom (0.1 per million inhabitants) (14). In Finland, an example of a sparsely populated country, 5 of all 333 hospitals met criteria for classification as comprehensive stroke centers (50). In 2011, 66% of Americans were within a 60 min ground transfer to a primary stroke center (51). However, only 56% of Americans had 60 min ground transfer proximity to a CSC (52). As a result, in some settings it is difficult to transport patients directly to CSC, especially with standard ambulance services. Population distribution and density are important to consider when planning stroke services.

### Onsite Triage to Avoid Secondary Transfer and In-Hospital Delays

Accurate triage and selection of the appropriate target hospital avoids the transfer of patients with large-vessel occlusion to hospitals without endovascular treatment services. It is estimated that every minute of delay in transfer reduces the probability that patients will receive intra-arterial treatment by 2.5% (53). Further, by identifying thrombectomy-eligible patients, appropriate triage prevents the overloading of comprehensive stroke centers.

The MSU strategy has been shown to be effective for the triage of stroke patients (54). CT-angiography has been used in MSUs to assess for LVO at the emergency site (19). Even use of non-contrast CT has been associated with reduction of delay before intra-arterial treatment (30). For patients with haemorrhagic stroke, MSU-based triage allows for transport to hospitals with neurosurgery services, bypassing hospitals without such capabilities (26). In the urban setting of Berlin, patients with hemorrhage transported to hospitals without neurosurgery services decreased from 43% in the conventional treatment group to 11% in the MSU group (43, 55). MSUs are also valuable for investigating other time-sensitive cerebral conditions such as traumatic brain injury or status epilepticus (56).

## Stroke Treatment Gaps for Rural Patients

For rural patients, distance and travel time to the nearest stroke center is a crucial issue for time-sensitive stroke treatment (57, 58). Times from symptom onset to rural hospital admission range from 5 to 30 h have been reported (59, 60). These transport delays contribute to the low rate of thrombolysis of 1–6% for patients in rural areas worldwide (61, 62). Further, rural-urban disparities in thrombolysis administration have increased in recent decades (63). This trend is seen not only in low and middle-income countries but also in high-income countries (59, 64, 65). In Australia, only 3% of rural patients were able to access stroke units in a timely manner compared to 77% of urban patients (66). In Canada, patients living in rural areas are less likely to receive stroke unit care, brain imaging within 24 h, carotid imaging, and neurologist consultations. Furthermore, rural patients were less likely to be transferred to inpatient rehabilitation facilities and less likely to receive physiotherapist, occupational therapist, and speech and language therapist review (67). In the United States, the Get With the Guidelines—Stroke Registry has identified the arrival to a rural hospital as one of the factors associated failure of thrombolytic therapy (68).

The treatment gap between urban and rural areas is even more pronounced with endovascular treatment options for acute stroke (69). For the provision of intra-arterial therapy, the center requires highly specialized staff (such as vascular neurologists, neurointensivists, neuroradiologists, and anaesthesiologists), capable facilities and technical resources. This complex infrastructure is only available in limited CSCs which are located almost exclusively in urban centers (70). Consequently, patients living in rural and remote areas have limited access to timely IAT services (71).

Several strategies have been implemented in an effort to increasing thrombolysis rates. Health system factors generally associated with higher thrombolysis rates are urban location, centralized or hub and spoke models, treatment by a neurologist/stroke nurse, in a neurology department/stroke unit or teaching hospital, being admitted by ambulance or mobile team and stroke-specific protocols (72). Thrombolysis rates may be dependent on the hospital's level of stroke service (with stroke centers having the highest rates and hospitals without stroke units having the lowest rates) and patient factors such as age and preexisting disabilities (15). Accordingly, organizational streamlining by bypassing hospitals without stroke units may be sensible. However, in sparsely populated areas with long distances to the nearest stroke unit, it may be reasonable to initiate thrombolysis at a local hospital (supported by telemedicine, if available) before transferring the patient to center with a higher level of stroke care (73).

## Adapting MSU Approaches to Rural Healthcare

MSUs provide a valuable resource to rural and remote settings where patient may not have easy access to in-hospital stroke care (74). The MSU model can be adapted to local settings based on local needs. Accessing telemedicine technologies through cellular communication provides instant information enabling healthcare providers to reach out beyond the doors of the hospital.

As MSUs typically look to integrate with local emergency response services, there are several models of MSU staff composition. On board personnel can include vascular neurologists, paramedics, nurses or radiographers (25). Staff composition can be adapted to address the need of rural settings. In Norway, MSU staffing and responsibilities have been developed to work together with the existing EMS framework for rural and remote health (32, 75–77). In these smaller urban areas, the MSU is staffed with an anaesthesiologist, a paramedic and a nurse paramedic. Anaesthesiologists have been trained to identify and treat stroke (76). The anaesthesiologist may also provide resuscitation and perform invasive emergency procedures to any unstable or critically ill patient (77). Evolving telestroke technology requires staff to have ongoing, intermittent or mock training (78).

Subsequent iterations of the MSU have adapted to their respective settings. Larger vehicles have the advantage of carrying larger scanners and more specialized personnel, robustness in rural off-road conditions, and allowing relatives to accompany patients to provide history and procedural consent (79). Smaller vehicles may have greater access to narrow roads and lower cost. With this in mind, vehicle models should be selected according to the specific needs of the region and healthcare setting (25).

In Australia, a potential solution to serving remote patients with an Air Mobile Stroke Unit (Air-MSU) is being explored (80). With this approach, the MSU concept is being extended to another transport vehicle by equipping a helicopter or airplane with the CT scanner, POCT, and telemedicine connectivity.

**Figure 3:**
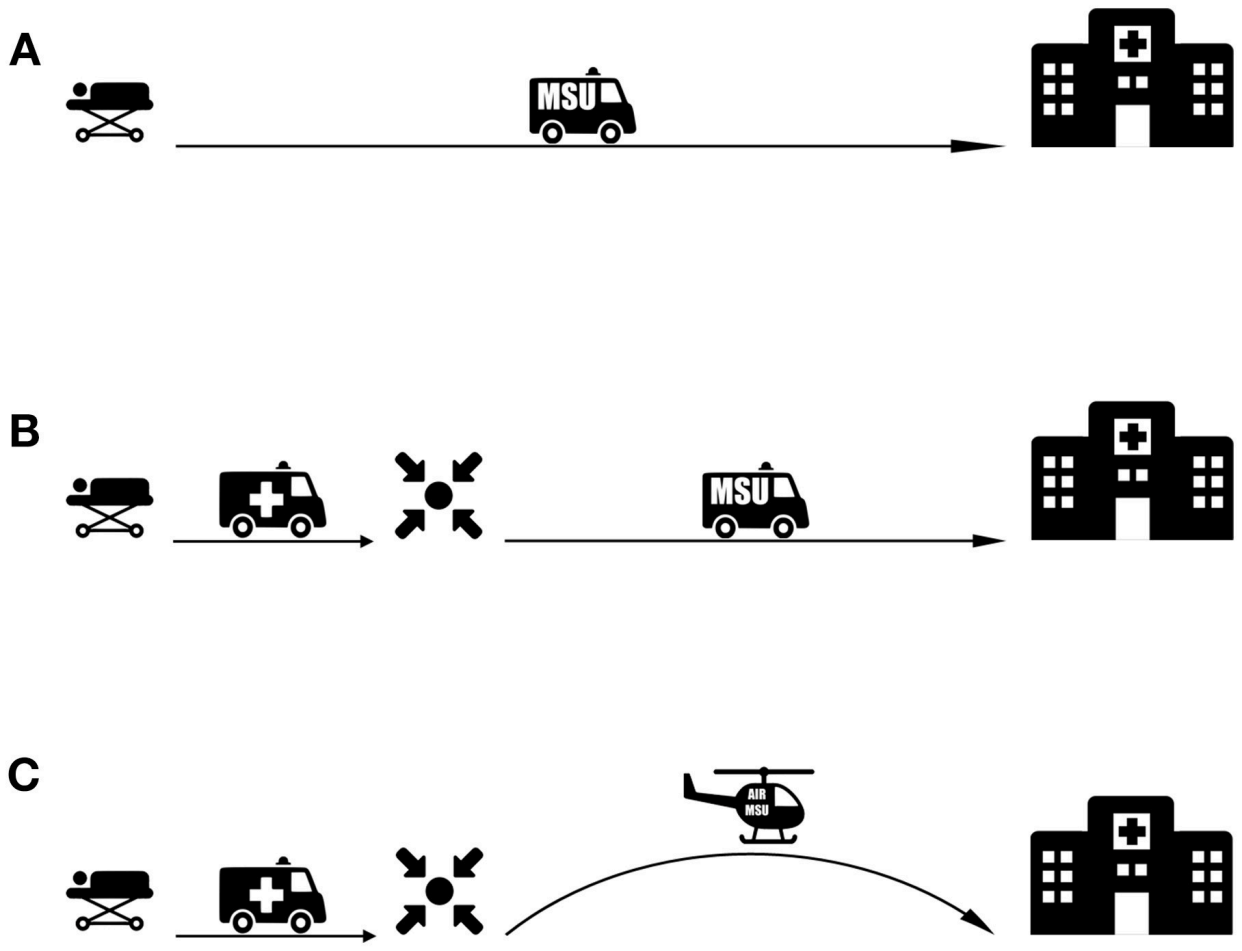
MSU-based transport strategies for patients with LVO. **(A)** An MSU-based model where imaging and triage is performed onsite and the patient is transported directly to a CSC. **(B)** Rendezvous approach extending MSU range for rural areas. A conventional ambulance transports the patient to a rendezvous point with the MSU. After MSU-based imaging and diagnosis, the patient is transported directly to a CSC. **(C)** Proposed Rendezvous approach with an Air-MSU suitable for remote areas with large transit distances.

A rendezvous model has been studied to enable the MSU cover large rural areas (Figure 3). With this approach, a conventional EMS ambulance is dispatched to the patient location and then travels toward the hospital. The EMS ambulance is met at predetermined rendezvous locations—approximately half the distance—by the MSU. Predetermined rendezvous points are selected based on factors including catchment area, ease of road access, road connectivity and wireless signal strength (35). In a large rural area in Northern Alberta, Canada, this rendezvous approach has increased the catchment area to a 250 km radius surrounding the Comprehensive Stroke Center (35). This approach can be adapted to urban areas close to the periphery of the MSUs direct response area, effectively increasing the catchment areas of these units.

For rural and remote patients, there are also opportunities for incorporation of the MSU concept with the air ambulance service. In these situations, the air ambulance can act as the first responding vehicle to meet and transport the patient. Here, the patient can be transported to a predetermined rendezvous point or to a PSC where the patient can be met by the MSU. For a proposed Air-MSU, the CT-equipped airplane may land at the emergency site, and undertake onsite specialized stroke care and imaging (80). Alternatively, conventional EMS (ground or air) can meet the patient at the emergency site and transport the patient to a rendezvous point or PSC where the patient is met by the Air-MSU.

## Cost Effectiveness

While the MSU strategy remains an innovative approach to prehospital acute stroke management, more data is required to best understand the costs of the MSU, its staffing, and operation. Two independent preliminary analyses of cost-effectiveness have reported encouraging results. Based on the Homburg trial, analysis demonstrated an improvement in cost-benefit with reduction of personnel and an optimal cost-benefit with an operating radius between 43.01 and 64.88 km (81). In an analysis of Berlin-based MSUs, a higher rate of thrombolysis and earlier treatment for MSU patients resulted in reduced disability and its associated costs (82). While both of these studies suggest cost benefits, prospective cost efficiency data comparing MSUs to standard management is expected and awaited from the BEST-MSU trial (83).

MSU cost-effectiveness may be improved by substituting onboard physicians for telemedicine-linked remote experts (84). Considering the onboard CT scanners, there is the possibility for increased demand and improved technology to help reduce this high startup cost as economies of scale take their effect (85). Future services may be expanded to include other cerebral emergencies and treatment modalities.

Considering rural and underserved areas, the cost impact of frequency of deployment, operational area, personnel costs and support or replacement of other services may differ from urban settings and trial scenarios. MSU programmes may involve single or multiple units to cover large geographic areas and may require additional co-ordination with local EMS if, for example, a rendezvous approach is taken. Payment and reimbursement models particular to a health care system would impact cost burden to emergency and hospital services (86). In some rural settings, ambulance agencies may not be directly affiliated with the relevant hospital impacting costs for the health services involved (74).

Future prospective research is required in defining costs for establishing and maintaining MSUs and costs for both acute and long-term care of patients managed both on MSUs and by standard emergency services. It is important that this financial cost is weighed with the perspective of important stakeholders in acute stroke care and consideration of total hospital and long-term care costs to the health care system for each stroke patient. As MSU configuration and operation is influenced strongly by setting, further health economic analyses in a variety of settings are required.

## Telemedicine for MSUs

Telecommunication approaches between MSUs and the stroke center via systems can provide real-time remote specialist advice by teleradiology (transmission of high-quality images) and telestroke assessment (real-time bidirectional videoconferencing and high-speed videos transmission) (25). As such, MSUs can provide advanced imaging and expert consultation at either the emergency site or at smaller healthcare centers which otherwise would not have this capability.

Teleradiology enables the transmission of images and information to physicians and specialists for use in remote diagnoses and medical consultation. Through MSU-based teleradiology high-resolution medical images (such as CT scans) can be obtained onsite, transmitted while stationary or en route, and interpreted by experts at a major university medical center.

Telestroke planning can be organized into a distributed network or hub-and-spoke model. In a distributed network model, the telestroke specialist is affiliated with a third party employer which provides contracted services with the originating hospital site (87). In a hub-and-spoke model, specialty care is provided to patients at community settings (spokes) by specialists affiliated with larger, more comprehensive tertiary care centers (hubs). Spokes are primary and secondary care centers which can be linked to distant sites—even more than 300 km away—where the telestroke provider is located. At hubs, vascular neurologists and other acute stroke specialists compose a call panel delivering telestroke services (78). Hubs further act as recipients for patients which require transfer to a higher level of care. Hub-and-spoke models have been shown to be cost-effective and to increase the catchment population (88, 89).

### Reliability of MSU-Based Telemedicine

Technical innovation in the transmission of data between the hospital and MSU plays an important role. Early studies encountered difficulties in telecommunication in part due to suboptimal 3G public network availability (90–94). Fortunately, with improved technology and 4G mobile systems telecommunication is becoming more reliable (25). Telemedicine encounters between the MSU and hospital have been shown to be successfully completed for 99% of patients with 4G connectivity (95). Low-cost, tablet-based platform via commercial cellular networks (4G/LTE) were used to reliably perform prehospital neurologic assessments (NIHSS) of actors in both rural (central Virginia) and urban settings (San Francisco Bay Area) via videoconferencing (96). With innovation it is important to ensure that bidirectional telecommunication is encrypted, secure and meets standards for transmission of protected health information.

The reliability of MSU-based telestroke assessment has been evaluated. Remote stroke assessment through telemedicine by a vascular neurologist has been shown to be reliable and comparable to in-person assessment (94). Further treatment decisions for thrombolysis showed strong agreement between an on-board vascular neurologist and a telemedicine vascular neurologist (84). The level of agreement is comparable to two vascular neurologists evaluating the same patients face-to-face in the emergency department (97). Importantly, the time to treatment decision and thrombolysis administration has been shown to be comparable between on-board and telemedicine vascular neurologists (98).

### Limitations

While MSU-based telestroke approaches are promising, there are limitations to the management tasks which can be carried out remotely. The treatment of acute stroke in an MSU is a complex exercise involving multiple parallel tasks being carried out by several healthcare professionals within the confined space of an MSU. This includes neurological assessment, monitoring of vital signs, patient positioning, management of patient comfort, and possible restraint, CT scanning, point-of-care laboratory testing, and medication preparation and administration (86). Furthermore, the clinical decision on whether to administer thrombolysis requires training, experience, and careful clinical judgment. As such, there may be a limit to clinical decisions which can be carried out in the physical absence of a vascular neurologist via telemedicine. Looking beyond the first hour's hyperacute care, stroke patients require ongoing care from physiotherapists, occupational therapists and speech and language therapists. When considering the extension of stroke care to rural and underserved areas with MSUs, further work is required to determine optimal models for integration of these services.

EMS response varies worldwide, including between many European and US MSU settings. In the United States, ambulances are typically not staffed by physicians. As a result, in developing an MSU programme, a decision has to be made as to whether to include an on board physician. Early experience from Houston suggests the ratio of MSU alerts from EMS dispatch to tPA treatments is up to 10 to 1, suggesting that it may be impractical to have a vascular neurologist aboard the MSU for all calls (86).

## Improving Prehospital Stroke Care in Low and Middle-Income Countries

With rapid advances in prehospital stroke care primarily in high-income countries (HIC), it is important to consider opportunities, challenges and the applicability of these approaches to low and-middle income countries (LMIC) as well. There is a growing disparity in the burden of stroke between LMIC and HIC. About 75% of deaths from stroke and more than 80% of disability-adjusted life years (DALYs) occur in LMICs (99–103). Further, in the last four decades, there has been a 42% decrease in stroke incidence in high-income countries and a 100% increase in LMIC (104). Cerebrovascular diseases in sub-Saharan Africa are increasingly frequent and associated with a poor outcome (2, 105–111). Unfortunately, there is very limited data on the organization of prehospital stroke services in such settings. For example, only 3 African countries (South Africa, Egypt, and Morocco) have reported experiences on thrombolysis (112).

There are multifactorial barriers to implementing effective prehospital stroke care in the LMIC setting. Prehospital barriers include unavailable/inadequate transportation and a lack of trained stroke specialists (101, 102, 113). Considering transport, in many cases, the ambulances are not well-equipped and do not have trained personnel (114). Further, most patients in these settings use their personal or hired vehicles, rather than ambulances, to seek medical help (113, 115). In these settings, patients who are transported by ambulances are predominantly those with trauma injuries and obstetric emergencies (113).

The global deficit in skilled healthcare personnel is most pronounced in rural areas, especially in LMIC (114, 116). In India, 80% of specialists live in urban areas. Consequently, 700 million people living in rural India have to travel a distance of 75 to 100 km for a tertiary consultation (117). There remains a paucity of neurologists worldwide, especially in the LMIC setting. The global median of adult neurologists is 0.43 per 100 000 population, with the number of adult neurologists ranging from 0.04 (in LIC) to 4.75 (in HIC) per 100,000 population (118). In India, nearly 1 billion people live in regions lacking access to a practicing neurologist (119).

With limited specialized personnel and transport available, telemedicine could be helpful to address gaps in stroke service delivery. Remote teleconsultations would allow the few, existing specialists, primarily situated in urban areas, to provide expertise to a greater number of patients who are situated in rural settings. To overcome infrastructure and connectivity hurdles, satellite-based telemedicine has been effectively employed in rural India (120).

A telemedicine-capable MSU could provide specialized care in the regions with limited hospital emergency departments and lacking EMS systems. MSUs could function as mobile clinics when not in use in emergencies. As such, they could provide hospital-caliber imaging and laboratory services to underserved areas.

Encouragingly there are a growing number of MSU programmes in LMIC settings. For example, in Thailand, a stroke fast track programme, telestroke, and two MSUs in Bangkok are improving stroke care (121). Inclusion of MSUs should be considered as part of long-term planning for stroke service improvement in these settings.

MSU operation is influenced strongly by setting—be it urban or rural, or high- or low-income. In metropolitan settings, factors such as traffic congestion and existing emergency response configurations as well as geo- and socio-spatial determinants of emergency service utilization impact transport modeling (122). In LMIC, road conditions impact both transport planning and may necessitate physical upgrades to the MSU vehicle. However, data in these settings remains limited. As the preponderance of MSU studies to date have been conducted in HIC, further research is required better understand the implementation and optimization of MSU transport models in the LMIC setting.

Considering the prehospital phase as a whole, there are alternative strategies for early identification and preclinical selection of stroke patients which may have applicability to rural patients and those in resource-limited settings. Several prehospital stroke screening scales have been developed and employed in an effort to assist dispatchers in identifying stroke patients with high specificity and sensitivity (123–125). Additionally, there are several prehospital scales assessing stroke severity with the potential to help identify patients with LVOs (126–128). Nevertheless, prehospital stroke scales vary in their accuracy and may be influenced by levels of stroke scale training and provider educational standards (124, 129). Public awareness campaigns, training of dispatchers, and paramedics are also effective methods of early identification of stroke which may be complementary to other prehospital strategies (130–135). The quality of stroke care varies across the world depending upon location, local hospital facilities, ability to pay, education, and cultural, social, or religious beliefs (136). As such, prehospital strategies should be tailored to local health needs, affordability and existing stroke services.

## Future Directions

Future MSU service planning may involve integration of multiple concepts. Further adaption will allow MSUs to better integrate into local and regional emergency medical services. Remote locations may eventually benefit from a combination of the Air MSU and rendezvous model. Specialized training of on board personnel may further alleviate the need for an on board vascular neurologist. Improvements in technology may allow for smaller, lighter and more robust units CT scanners. The MSU concept can be expanded to treat other emergencies in underserved areas. Further studies are required to better understand the medical and health-economic benefit of each model.

## Author Contributions

SM and KF contributed to the conception and design of the work, literature search, analysis and interpretation, and article drafting. SM, SW, IQG, SAH, ML, and KF contributed to critical revision. All authors gave final approval of the version to be published.

### Conflict of Interest Statement

The authors declare that the research was conducted in the absence of any commercial or financial relationships that could be construed as a potential conflict of interest.

## References

[1] Norrving B Kissela B. The global burden of stroke and need for a continuum of care. Neurology. (2013) 80(3 Suppl. 2):S5–12. 10.1212/WNL.0b013e3182762397 23319486 PMC12443346

[2] Feigin VL Forouzanfar MH Krishnamurthi R Mensah GA Connor M Bennett DA. Global and regional burden of stroke during 1990–2010: findings from the Global Burden of Disease Study 2010. Lancet. (2014) 383:245–54. 10.1016/S0140-6736(13)61953-4 24449944 PMC4181600

[3] Ovbiagele B Goldstein LB Higashida RT Howard VJ Johnston SC Khavjou OA. Forecasting the future of stroke in the United States: a policy statement from the American Heart Association and American Stroke Association. Stroke. (2013) 44:2361–75. 10.1161/STR.0b013e31829734f2 23697546

[4] Kobayashi A Czlonkowska A Ford GA Fonseca AC Luijckx GJ Korv J. European Academy of Neurology and European Stroke Organization consensus statement and practical guidance for pre-hospital management of stroke. Eur J Neurol. (2018) 25:425–33. 10.1111/ene.13539 29218822

[5] Powers WJ Rabinstein AA Ackerson T Adevoe OM Bambakidis NC Becker K. 2018 Guidelines for the early management of patients with acute ischemic stroke: a guideline for healthcare professionals from the American Heart Association/American Stroke Association. Stroke. (2018) 49:e46–110. 10.1161/STR.0000000000000158 29367334

[6] Saver JL. Time is brain–quantified. Stroke. (2006) 37:263–6. 10.1161/01.STR.0000196957.55928.ab 16339467

[7] Saver JL Fonarow GC Smith EE Reeves MJ Grau-Sepulveda MV Pan W. Time to treatment with intravenous tissue plasminogen activator and outcome from acute ischemic stroke. JAMA. (2013) 309:2480–8. 10.1001/jama.2013.6959 23780461

[8] Strbian D Soinne L Sairanen T Häppölä O Lindsberg PJ Tatlisumak T. Ultraearly thrombolysis in acute ischemic stroke is associated with better outcome and lower mortality. Stroke. (2010) 41:712–6. 10.1161/STROKEAHA.109.571976 20167917

[9] Goyal M Menon BK van Zwam WH Dippel DW Mitchell PJ Demchuk AM. Endovascular thrombectomy after large-vessel ischaemic stroke: a meta-analysis of individual patient data from five randomised trials. Lancet. (2016) 387:1723–31. 10.1016/S0140-6736(16)00163-X 26898852

[10] Khatri P Yeatts SD Mazighi M Broderick JP Liebeskind DS Demchuk AM. Time to angiographic reperfusion and clinical outcome after acute ischaemic stroke: an analysis of data from the Interventional Management of Stroke (IMS III) phase 3 trial. Lancet Neurol. (2014) 13:567–74. 10.1016/S1474-4422(14)70066-3 24784550 PMC4174410

[11] Sun CH Ribo M Goyal M Yoo AJ Jovin T Cronin CA. Door-to-puncture: a practical metric for capturing and enhancing system processes associated with endovascular stroke care, preliminary results from the rapid reperfusion registry. J Am Heart Assoc. (2014) 3:e000859. 10.1161/JAHA.114.000859 24772523 PMC4187502

[12] Adeoye O Hornung R Khatri P Kleindorfer D. Recombinant tissue-type plasminogen activator use for ischemic stroke in the United States: a doubling of treatment rates over the course of 5 years. Stroke. (2011) 42:1952–5. 10.1161/STROKEAHA.110.612358 21636813 PMC4114342

[13] Schwamm LH Ali SF Reeves MJ Smith EE Saver JL Messe S. Temporal trends in patient characteristics and treatment with intravenous thrombolysis among acute ischemic stroke patients at Get With The Guidelines-Stroke hospitals. Circ Cardiovasc Qual Outcomes. (2013) 6:543–9. 10.1161/CIRCOUTCOMES.111.000095 24046398

[14] Aguiar de Sousa D von Martial R Abilleira S Gattringer T Kobayashi A Gallofré M Access to and delivery of acute ischaemic stroke treatments: a survey of national scientific societies and stroke experts in 44 European countries. Eur Stroke J. (2018) 1–16. 10.1177/2396987318786023 PMC6533860 31165091

[15] Gumbinger C Reuter B Hacke W Sauer T Bruder I Diehm C. Restriction of therapy mainly explains lower thrombolysis rates in reduced stroke service levels. Neurology. (2016) 86:1975–83. 10.1212/WNL.0000000000002695 27164674

[16] Messé SR Fonarow GC Smith EE Kaltenbach L Olson DM Kasner SE. Use of tissue-type plasminogen activator before and after publication of the European Cooperative Acute stroke study III in get with the guidelines-stroke. Circ Cardiovasc Qual Outcomes. (2012) 5:321–6. 10.1161/CIRCOUTCOMES.111.964064 22550132

[17] Agyeman O Nedeltchev K Arnold M Fischer U Remonda L Isenegger J. Time to admission in acute ischemic stroke and transient ischemic attack. Stroke. (2006) 37:963–6. 10.1161/01.STR.0000206546.76860.6b 16514096

[18] Evenson KR Foraker RE Morris DL Rosamond WD. A comprehensive review of prehospital and in-hospital delay times in acute stroke care. Int J Stroke. (2009) 4:187–99. 10.1111/j.1747-4949.2009.00276.x 19659821 PMC2825147

[19] Walter S Kostopoulos P Haass A Keller I Lesmeister M Schlechtriemen T. Diagnosis and treatment of patients with stroke in a mobile stroke unit versus in hospital: a randomised controlled trial. Lancet Neurol. (2012) 11:397–404. 10.1016/S1474-4422(12)70057-1 22497929

[20] Fassbender K Walter S Liu Y Muehlhauser F Ragoschke A Kuehl S. “Mobile stroke unit” for hyperacute stroke treatment. Stroke. (2003) 34:e44. 10.1161/01.STR.0000075573.22885.3B 12750527

[21] Walter S Kostopoulos P Haass A Lesmeister M Grasu M Grunwald I. Point-of-care laboratory halves door-to-therapy-decision time in acute stroke. Ann Neurol. (2011) 69:581–6. 10.1002/ana.22355 21400566

[22] Kettner M Helwig SA Ragoschke-Schumm A Schwindling L Roumia S Keller I. Prehospital computed tomography angiography in acute stroke management. Cerebrovasc Dis. (2017) 44:338–43. 10.1159/000484097 29130951

[23] Audebert H Fassbender K Hussain MS Ebinger M Turc G Uchino K. The PRE-hospital stroke treatment organization. Int J Stroke. (2017) 12:932–40. 10.1177/1747493017729268 28872449

[24] Fassbender K Balucani C Walter S Levine SR Haass A Grotta J. Streamlining of prehospital stroke management: the golden hour. Lancet Neurol. (2013) 12:585–96. 10.1016/S1474-4422(13)70100-5 23684084

[25] Fassbender K Grotta JC Walter S Grunwald IQ Ragoschke-Schumm A Saver JL. Mobile stroke units for prehospital thrombolysis, triage, and beyond: benefits and challenges. Lancet Neurol. (2017) 16:227–37. 10.1016/S1474-4422(17)30008-X 28229894

[26] Kostopoulos P Walter S Haass A Papanagiotou P Roth C Yilmaz U. Mobile stroke unit for diagnosis-based triage of persons with suspected stroke. Neurology. (2012) 78:1849–52. 10.1212/WNL.0b013e318258f773 22592363

[27] Walter S Kostpopoulos P Haass A Helwig S Keller I Licina T. Bringing the hospital to the patient: first treatment of stroke patients at the emergency site. PLoS ONE. (2010) 5:e13758. 10.1371/journal.pone.0013758 21060800 PMC2966432

[28] Ebinger M Winter B Wendt M Weber JE Waldschmidt C Rozanski M. Effect of the use of ambulance-based thrombolysis on time to thrombolysis in acute ischemic stroke: a randomized clinical trial. JAMA. (2014) 311:1622–31. 10.1001/jama.2014.2850 24756512

[29] Bowry R Parker S Rajan SS Yamal JM Wu TC Richardson L. Benefits of stroke treatment using a mobile stroke unit compared with standard management: the BEST-MSU study run-in phase. Stroke. (2015) 46:3370–4. 10.1161/STROKEAHA.115.011093 26508753

[30] Cerejo R John S Buletko AB Taqui A Itrat A Organek N. A mobile stroke treatment unit for field triage of patients for intraarterial revascularization Therapy. J Neuroimaging. (2015) 25:940–5. 10.1111/jon.12276 26179631

[31] Parker SA Bowry R Wu TC Noser EA Jackson K Richardson L. Establishing the first mobile stroke unit in the United States. Stroke. (2015) 46:1384–91. 10.1161/STROKEAHA.114.007993 25782464

[32] Hov MR Zakariassen E Lindner T Nome T Bache KG Røislien J. Interpretation of brain CT scans in the field by critical care physicians in a mobile stroke unit. J Neuroimaging. (2018) 28:106–11. 10.1111/jon.12458 28766306 PMC5811888

[33] Kummer BR Lerario MP Navi BB Ganzman AC Ribaudo D Mir SA. Clinical information systems integration in New York city's first mobile stroke unit. Appl Clin Informatics. (2018) 9:89–98. 10.1055/s-0037-1621704 29415308 PMC5802999

[34] Lin E Calderon V Goins-Whitmore J Bansal V Zaidat O. World's first 24/7 mobile stroke unit: initial 6-month experience at mercy health in Toledo, Ohio. Front Neurol. (2018) 9:283. 10.3389/fneur.2018.00283 29867711 PMC5966532

[35] Shuaib A Jeerakathil T Alberta Mobile Stroke Unit I. The mobile stroke unit and management of acute stroke in rural settings. CMAJ. (2018) 190:E855–8. 10.1503/cmaj.170999 30012801 PMC6050120

[36] Audebert HJ Saver JL Starkman S Lees KR Endres M. Prehospital stroke care: new prospects for treatment and clinical research. Neurology. (2013) 81:501–8. 10.1212/WNL.0b013e31829e0fdd 23897876 PMC3776535

[37] Kim JT Fonarow GC Smith EE Reeves MJ Navalkele DD Grotta JC. Treatment with tissue plasminogen activator in the golden hour and the shape of the 4.5-hour time-benefit curve in the National United States get with the guidelines-stroke population. Circulation. (2017) 135:128–39. 10.1161/CIRCULATIONAHA.116.023336 27815374

[38] Kunz A Nolte CH Erdur H Fiebach JB Geisler F Rozanski M. Effects of ultraearly intravenous thrombolysis on outcomes in ischemic stroke: the STEMO (Stroke Emergency Mobile) Group. Circulation. (2017) 135:1765–7. 10.1161/CIRCULATIONAHA.117.027693 28461420

[39] Kunz A Ebinger M Geisler F Rozanski M Waldschmidt C Weber JE. Functional outcomes of pre-hospital thrombolysis in a mobile stroke treatment unit compared with conventional care: an observational registry study. Lancet Neurol. (2016) 15:1035–43. 10.1016/S1474-4422(16)30129-6 27430529

[40] Nolte CH Ebinger M Scheitz JF Kunz A Erdur H Geisler F. Effects of prehospital thrombolysis in stroke patients with prestroke dependency. Stroke. (2018) 49:646–51. 10.1161/STROKEAHA.117.019060 29459395

[41] Tsivgoulis G Geisler F Katsanos AH Kõrv J Kunz A Mikulik R. Ultraearly intravenous thrombolysis for acute ischemic stroke in mobile stroke unit and hospital settings. Stroke. (2018) 49:1996–9. 10.1161/STROKEAHA.118.021536 29986934

[42] Taqui A Cerejo R Itrat A Briggs FB Reimer AP Winners S. Reduction in time to treatment in prehospital telemedicine evaluation and thrombolysis. Neurology. (2017) 88:1305–12. 10.1212/WNL.0000000000003786 28275084

[43] Ebinger M Kunz A Wendt M Rozanski M Winter B Waldschmidt C. Effects of golden hour thrombolysis: a Prehospital Acute Neurological Treatment and Optimization of Medical Care in Stroke (PHANTOM-S) substudy. JAMA Neurol. (2015) 72:25–30. 10.1001/jamaneurol.2014.3188 25402214

[44] Gorelick PB. Primary and comprehensive stroke centers: history, value and certification criteria. J Stroke. (2013) 15:78–89. 10.5853/jos.2013.15.2.78 24324943 PMC3779669

[45] Alberts MJ Hademenos G Latchaw RE Jagoda A Marler JR Mayberg MR. Recommendations for the establishment of primary stroke centers. Brain Attack Coalition. JAMA. (2000) 283:3102–9. 10.1001/jama.283.23.3102 10865305

[46] Alberts MJ Latchaw RE Selman WR Shephard T Hadley MN Brass LM. Recommendations for comprehensive stroke centers: a consensus statement from the Brain Attack Coalition. Stroke. (2005) 36:1597–616. 10.1161/01.STR.0000170622.07210.b4 15961715

[47] Gerschenfeld G Muresan IP Blanc R Obadia M Abrivard M Piotin M. Two paradigms for endovascular thrombectomy after intravenous thrombolysis for acute ischemic stroke. JAMA Neurol. (2017) 74:549–56. 10.1001/jamaneurol.2016.5823 28319240 PMC5822198

[48] Badhiwala JH Nassiri F Alhazzani W Selim MH Farrokhyar F Spears J. Endovascular thrombectomy for acute ischemic stroke: a meta-analysis. JAMA. (2015) 314:1832–43. 10.1001/jama.2015.13767 26529161

[49] Zaidat OO Yoo AJ Khatri P Tomsick TA von Kummer R Saver JL. Recommendations on angiographic revascularization grading standards for acute ischemic stroke: a consensus statement. Stroke. (2013) 44:2650–63. 10.1161/STROKEAHA.113.001972 23920012 PMC4160883

[50] Meretoja A Roine RO Kaste M Linna M Juntunen M Erilä T. Stroke monitoring on a national level: PERFECT Stroke, a comprehensive, registry-linkage stroke database in Finland. Stroke. (2010) 41:2239–46. 10.1161/STROKEAHA.110.595173 20798363

[51] Mullen MT Wiebe DJ Bowman A Wolff CS Albright KC Roy J. Disparities in accessibility of certified primary stroke centers. Stroke. (2014) 45:3381–8. 10.1161/STROKEAHA.114.006021 25300972 PMC4282182

[52] Adeoye O Albright KC Carr BG Wolff C Mullen MT Abruzzo T. Geographic access to acute stroke care in the United States. Stroke. (2014) 45:3019–24. 10.1161/STROKEAHA.114.006293 25158773 PMC5877807

[53] Prabhakaran S Ward E John S Lopes DK Chen M Temes RE. Transfer delay is a major factor limiting the use of intra-arterial treatment in acute ischemic stroke. Stroke. (2011) 42:1626–30. 10.1161/STROKEAHA.110.609750 21527756

[54] Wendt M Ebinger M Kunz A Rozanski M Waldschmidt C Weber JE. Improved prehospital triage of patients with stroke in a specialized stroke ambulance: results of the pre-hospital acute neurological therapy and optimization of medical care in stroke study. Stroke. (2015) 46:740–5. 10.1161/STROKEAHA.114.008159 25634000

[55] Ebinger M Rozanski M Waldschmidt C Weber J Wendt M Winter B. PHANTOM-S: the prehospital acute neurological therapy and optimization of medical care in stroke patients—study. Int J Stroke. (2012) 7:348–53. 10.1111/j.1747-4949.2011.00756.x 22300008

[56] Schwindling L Ragoschke-Schumm A Kettner M Helwig S Manitz M Roumia S. Prehospital imaging-based triage of head trauma with a mobile stroke unit: first evidence and literature review. J Neuroimaging. (2016) 26:489–93. 10.1111/jon.12355 27159772

[57] Cox AM McKevitt C Rudd AG Wolfe CD. Socioeconomic status and stroke. Lancet Neurol. (2006) 5:181–8. 10.1016/S1474-4422(06)70351-9 16426994

[58] Teuschl Y Brainin M. Stroke education: discrepancies among factors influencing prehospital delay and stroke knowledge. Int J Stroke. (2010) 5:187–208. 10.1111/j.1747-4949.2010.00428.x 20536616

[59] Joubert J Prentice LF Moulin T Liaw ST Joubert LB Preux PM. Stroke in rural areas and small communities. Stroke. (2008) 39:1920–8. 10.1161/STROKEAHA.107.501643 18420955

[60] Oliveira-Filho J Martins SC Pontes-Neto OM Longo A Evaristo EF Carvalho JJ Guidelines for acute ischemic stroke treatment: part I. Arq Neuro Psiquiatr. (2012) 70:621–9. 10.1590/S0004-282X2012000800012 22899035

[61] Cadilhac DA Purvis T Kilkenny MF Longworth M Mohr K Pollack M. Evaluation of rural stroke services: does implementation of coordinators and pathways improve care in rural hospitals? Stroke. (2013) 44:2848–53. 10.1161/STROKEAHA.113.001258 23950561

[62] Newbury J Kleinig T Leyden J Arima H Castle S Cranefield J. Stroke Epidemiology in an Australian Rural Cohort (SEARCH). Int J Stroke. (2017) 12:161–8. 10.1177/1747493016670174 27694313

[63] Gonzales S Mullen MT Skolarus L Thibault DP Udoeyo U Willis AW. Progressive rural-urban disparity in acute stroke care. Neurology. (2017) 88:441–8. 10.1212/WNL.0000000000003562 28053009 PMC5278944

[64] Kozera G Chwojnicki K Gójska-Grymajło A Gasecki D Schminke U Nyka WM. Pre-hospital delays and intravenous thrombolysis in urban and rural areas. Acta Neurol Scandin. (2012) 126:171–7. 10.1111/j.1600-0404.2011.01616.x 22077692

[65] Nasr DM Brinjikji W Cloft HJ Rabinstein AA. Utilization of intravenous thrombolysis is increasing in the United States. Int J Stroke. (2013) 8:681–8. 10.1111/j.1747-4949.2012.00844.x 22882725

[66] Eissa A Krass I Bajorek BV. Optimizing the management of acute ischaemic stroke: a review of the utilization of intravenous recombinant tissue plasminogen activator (tPA). J Clin Pharm Therapeut. (2012) 37:620–9. 10.1111/j.1365-2710.2012.01366.x 22708668

[67] Koifman J Hall R Li S Stamplecoski M Fang J Saltman AP. The association between rural residence and stroke care and outcomes. J Neurol Sci. (2016) 363:16–20. 10.1016/j.jns.2016.02.019 27000213

[68] Messe SR Khatri P Reeves MJ Smith EE Saver JL Bhatt DL Why are acute ischemic stroke patients not receiving IV tPA? Results from a national registry. Neurology. (2016) 87:1565–74. 10.1212/WNL.0000000000003198 27629092 PMC5067546

[69] Prabhakaran S Ruff I Bernstein RA. Acute stroke intervention: a systematic review. JAMA. (2015) 313:1451–62. 10.1001/jama.2015.3058 25871671

[70] Menon BK Campbell BC Levi C Goyal M. Role of imaging in current acute ischemic stroke workflow for endovascular therapy. Stroke. (2015) 46:1453–61. 10.1161/STROKEAHA.115.009160 25944319

[71] Suzuki S Saver JL Scott P Jahan R Duckwiler G Starkman S. Access to intra-arterial therapies for acute ischemic stroke: an analysis of the US population. Am J Neuroradiol. (2004) 25:1802–6. 15569751 PMC8148724

[72] Paul CL Ryan A Rose S Attia JR Kerr E Koller C. How can we improve stroke thrombolysis rates? A review of health system factors and approaches associated with thrombolysis administration rates in acute stroke care. Implement Sci. (2016) 11:51. 10.1186/s13012-016-0414-6 27059183 PMC4825073

[73] Kaste M. Comment: Well-organized stroke service reduces the burden of stroke. Neurology. (2016) 86:1981. 10.1212/WNL.0000000000002709 27164688

[74] Southerland AM Brandler ES. The cost-efficiency of mobile stroke units: where the rubber meets the road. Neurology. (2017) 88:1300–1. 10.1212/WNL.0000000000003833 28275085

[75] Hov MR Nome T Zakariassen E Russell D Røislien J Lossius HM. Assessment of acute stroke cerebral CT examinations by anaesthesiologists. Acta Anaesthesiol Scand. (2015) 59:1179–86. 10.1111/aas.12542 25976840 PMC5029598

[76] Hov MR Røislien J Lindner T Zakariassen E Bache KCG Solyga VM. Stroke severity quantification by critical care physicians in a mobile stroke unit. Eur J Emerg Med. (2017). 10.1097/mej.0000000000000529. [Epub ahead of print]. 29239899 PMC6504122

[77] Hov MR Ryen A Finsnes K Storflor J Lindner T Gleditsch J. Pre-hospital ct diagnosis of subarachnoid hemorrhage. Scand J Trauma Resuscit Emerg Med. (2017) 25:21. 10.1186/s13049-017-0365-1 28245880 PMC5331704

[78] Demaerschalk BM Berg J Chong BW Gross H Nystrom K Adeoye O. American Telemedicine Association: telestroke guidelines. Telemed J E-Health. (2017) 23:376–89. 10.1089/tmj.2017.0006 28384077 PMC5802246

[79] Ashkenazi L Toledano R Novack V EIluz E Abu-Salamae I Ifergane G. Emergency department companions of stroke patients: implications on quality of care. Medicine. (2015) 94:e520. 10.1097/MD.0000000000000520 25738470 PMC4553959

[80] Walter S Zhao H Easton D Bil C Sauer J Liu Y. Air-Mobile Stroke Unit for access to stroke treatment in rural regions. Int J Stroke. (2018) 13:568–75. 10.1177/1747493018784450 30071807

[81] Dietrich M Walter S Ragoschke-Schumm A Helwig S Levine S Balucani C. Is prehospital treatment of acute stroke too expensive? An economic evaluation based on the first trial. Cerebrovasc Dis. (2014) 38:457–63. 10.1159/000371427 25531507

[82] Gyrd-Hansen D Olsen KR Bollweg K Kronborg C Ebinger M Audebert HJ. Cost-effectiveness estimate of prehospital thrombolysis: results of the PHANTOM-S study. Neurology. (2015) 84:1090–7. 10.1212/WNL.0000000000001366 25672925

[83] Yamal JM Rajan SS Parker SA Jacob AP Gonzalez MO Gonzales NR. Benefits of stroke treatment delivered using a mobile stroke unit trial. Int J Stroke. (2018) 13:321–7. 10.1177/1747493017711950 28612680

[84] Wu TC Parker SA Jagolino A Yamal JM Bowry R Thomas A. Telemedicine can replace the neurologist on a mobile stroke unit. Stroke. (2017) 48:493–6. 10.1161/STROKEAHA.116.015363 28082671

[85] Walter S Grunwald IQ Helwig SA Ragoschke-Schumm A Kettner M Fousse M Mobile stroke units—cost-effective or just an expensive hype? Curr Atheroscler Rep. (2018) 20:49 10.1007/s11883-018-0751-9 30159610

[86] Rajan SS Baraniuk S Parker S Wu TC Bowry R Grotta JC. Implementing a mobile stroke unit program in the United States: why, how, and how much? JAMA Neurol. (2015) 72:229–34. 10.1001/jamaneurol.2014.3618 25485723

[87] Wechsler LR Demaerschalk BM Schwamm LH Adeoye OM Audebert HJ Fanale CV. Telemedicine quality and outcomes in stroke: a scientific statement for healthcare professionals from the American Heart Association/American Stroke Association. Stroke. (2017) 48:e3–25. 10.1161/STR.0000000000000114 27811332

[88] Demaerschalk BM Kiernan TE Investigators S. Vascular neurology nurse practitioner provision of telemedicine consultations. Int J Telemed Appl. (2010) 2010:507071. 10.1155/2010/507071 20811594 PMC2929495

[89] Grotta JC Savitz SI Persse D. Stroke severity as well as time should determine stroke patient triage. Stroke. (2013) 44:555–7. 10.1161/STROKEAHA.112.669721 23287779

[90] Bergrath S Reich A Rossaint R Rörtgen D Gerber J Fischermann H. Feasibility of prehospital teleconsultation in acute stroke–a pilot study in clinical routine. PLoS ONE. (2012) 7:e36796. 10.1371/journal.pone.0036796 22629331 PMC3356340

[91] Eadie L Regan L Mort A Shannon H Walker J MacAden A. Telestroke assessment on the move: prehospital streamlining of patient pathways. Stroke. (2015) 46:e38–40. 10.1161/STROKEAHA.114.007475 25550375

[92] Liman TG Winter B Waldschmidt C Zerbe N Hufnagl P Audebert HJ. Telestroke ambulances in prehospital stroke management: concept and pilot feasibility study. Stroke. (2012) 43:2086–90. 10.1161/STROKEAHA.112.657270 22693132

[93] Van Hooff RJ Cambron M Van Dyck R De Smedt A Moens M Espinoza AV. Prehospital unassisted assessment of stroke severity using telemedicine: a feasibility study. Stroke. (2013) 44:2907–9. 10.1161/STROKEAHA.113.002079 23920013

[94] Wu TC Nguyen C Ankrom C Yang J Persse D Vahidy F. Prehospital utility of rapid stroke evaluation using in-ambulance telemedicine: a pilot feasibility study. Stroke. (2014) 45:2342–7. 10.1161/STROKEAHA.114.005193 24938842 PMC4116449

[95] Itrat A Taqui A Cerejo R Briggs F Cho SM Organek N. Telemedicine in prehospital stroke evaluation and thrombolysis: taking stroke treatment to the doorstep. JAMA Neurol. (2016) 73:162–8. 10.1001/jamaneurol.2015.3849 26641366

[96] Chapman Smith SN Govindarajan P Padrick MM Lippman JM McMurry TL Resler BL. A low-cost, tablet-based option for prehospital neurologic assessment: the iTREAT study. Neurology. (2016) 87:19–26. 10.1212/WNL.0000000000002799 27281534 PMC4932237

[97] Ramadan AR Denny MC Vahidy F Yamal JM Wu TC Sarraj A. Agreement among stroke faculty and fellows in treating ischemic stroke patients with tissue-type plasminogen activator and thrombectomy. Stroke. (2017) 48:222–4. 10.1161/STROKEAHA.116.015214 27879445

[98] Bowry R Parker SA Yamal JM Hwang H Appana S Rangel-Gutierrez N. Time to decision and treatment with tPA (tissue-type plasminogen activator) using telemedicine versus an onboard neurologist on a mobile stroke unit. Stroke. (2018) 49:1528–30. 10.1161/STROKEAHA.117.020585 29720439

[99] Hata J Kiyohara Y. Epidemiology of stroke and coronary artery disease in Asia. Circ J. (2013) 77:1923–32. 10.1253/circj.CJ-13-0786 23842096

[100] Feigin VL Roth GA Naghavi M Parmar P Krishnamurthi R Chugh S. Global burden of stroke and risk factors in 188 countries, during 1990–2013: a systematic analysis for the Global Burden of Disease Study 2013. Lancet Neurol. (2016) 15:913–24. 10.1016/S1474-4422(16)30073-4 27291521

[101] Pandian JD Gall SL Kate MP Silva GS Akinyemi RO Ovbiagele BI. Prevention of stroke: a global perspective. Lancet. (2018) 392:1269–78. 10.1016/S0140-6736(18)31269-8 30319114

[102] Ghandehari K. Barriers of thrombolysis therapy in developing countries. Stroke Res Treatment. (2011) 2011:686797. 10.4061/2011/686797 21603174 PMC3095908

[103] Jaiteh LES Helwig SA Jagne A Ragoschke-Schumm A Sarr C Walter S. Standard operating procedures improve acute neurologic care in a sub-Saharan African setting. Neurology. (2017) 89:144–52. 10.1212/WNL.0000000000004080 28600460 PMC5501932

[104] Feigin VL Norrving B. A new paradigm for primary prevention strategy in people with elevated risk of stroke. Int J Stroke. (2014) 9:624–6. 10.1111/ijs.12300 24909195 PMC4140602

[105] Bower JH Asmera J Zebenigus M Sandroni P Bower SM Zenebe G. The burden of inpatient neurologic disease in two Ethiopian hospitals. Neurology. (2007) 68:338–42. 10.1212/01.wnl.0000252801.61190.e8 17261679

[106] Walker R Whiting D Unwin N Mugusi F Swai M Aris E. Stroke incidence in rural and urban Tanzania: a prospective, community-based study. Lancet Neurol. (2010) 9:786–92. 10.1016/S1474-4422(10)70144-7 20609629

[107] Walker RW McLarty DG Kitange HM Whiting D Masuki G Mtasiwa DM. Stroke mortality in urban and rural Tanzania. Adult Morbidity and Mortality Project. Lancet. (2000) 355:1684–7. 10.1016/S0140-6736(00)02240-6 10905244

[108] Walker RW Rolfe M Kelly PJ George MO James OF. Mortality and recovery after stroke in the Gambia. Stroke. (2003) 34:1604–9. 10.1161/01.STR.0000077943.63718.67 12817107

[109] Garbusinski JM van der Sande MA Bartholome EJ Dramaix M Gaye A Coleman R. Stroke presentation and outcome in developing countries: a prospective study in the Gambia. Stroke. (2005) 36:1388–93. 10.1161/01.STR.0000170717.91591.7d 15947255

[110] Connor MD Walker R Modi G Warlow CP. Burden of stroke in black populations in sub-Saharan Africa. Lancet Neurol. (2007) 6:269–78. 10.1016/S1474-4422(07)70002-9 17303533

[111] O'Donnell MJ Xavier D Liu L Zhang H Chin SL Rao-Melacini P. Risk factors for ischaemic and intracerebral haemorrhagic stroke in 22 countries (the INTERSTROKE study): a case-control study. Lancet. (2010) 376:112–23. 10.1016/S0140-6736(10)60834-3 20561675

[112] Akinyemi RO Adeniji OA Stroke care services in Africa: a systematic review. J Stroke Med. (2018) 1:55–64. 10.1177/2516608518775233

[113] Pandian JD William AG Kate MP Norrving B Mensah GA Davis S. Strategies to improve stroke care services in low- and middle-income countries: a systematic review. Neuroepidemiology. (2017) 49:45–61. 10.1159/000479518 28848165

[114] Strasser R Kam SM Regalado SM. Rural health care access and policy in developing countries. Ann Rev Publ Health. (2016) 37:395–412. 10.1146/annurev-publhealth-032315-021507 26735432

[115] Pandian JD Kalra G Jaison A Deepak SS Shamsher S Padala S. Factors delaying admission to a hospital-based stroke unit in India. J Stroke Cerebrovasc Dis. (2006) 15:81–7. 10.1016/j.jstrokecerebrovasdis.2006.01.001 17904057

[116] O'Donnell O. Access to health care in developing countries: breaking down demand side barriers. Cad Saude Publ. (2007) 23:2820–34. 10.1590/S0102-311X2007001200003 18157324

[117] Ganapathy K. Distribution of neurologists and neurosurgeons in India and its relevance to the adoption of telemedicine. Neurol India. (2015) 63:142–54. 10.4103/0028-3886.156274 25947977

[118] World Federation of Neurology World Health Organization ATLAS: Country Resources for Neurological Disorders, 2nd Ed. Geneva: Department of Mental Health and Substance Abuse (2017).

[119] Dorsey ER Glidden AM Holloway MR Birbeck GL Schwamm LH. Teleneurology and mobile technologies: the future of neurological care. Nat Rev Neurol. (2018) 14:285–97. 10.1038/nrneurol.2018.31 29623949

[120] Bagchi S. Telemedicine in rural India. PLoS Med. (2006) 3:e82. 10.1371/journal.pmed.0030082 16509768 PMC1420376

[121] Suwanwela NC Chutinet A Kijpaisalratana N Thrombolytic treatment in Thailand. J Stroke Med. (2018) 1:41–4. 10.1177/2516608518777934

[122] Rhudy JP Jr Alexandrov AW Rike J Bryndziar T Hossein Zadeh Maleki A Swatzell V. Geospatial visualization of mobile stroke unit dispatches: a method to optimize service performance. Interv Neurol. (2018) 7:464–70. 10.1159/000490581 30410526 PMC6216787

[123] Nor AM Davis J Sen B Shipsey D Louw SJ Dyker AG. The Recognition of Stroke in the Emergency Room (ROSIER) scale: development and validation of a stroke recognition instrument. Lancet Neurol. (2005) 4:727–34. 10.1016/S1474-4422(05)70201-5 16239179

[124] Brandler ES Sharma M Sinert RH Levine SR. Prehospital stroke scales in urban environments: a systematic review. Neurology. (2014) 82:2241–9. 10.1212/WNL.0000000000000523 24850487 PMC4113467

[125] Krebes S Ebinger M Baumann AM Kellner PA Rozanski M Doepp F. Development and validation of a dispatcher identification algorithm for stroke emergencies. Stroke. (2012) 43:776–81. 10.1161/STROKEAHA.111.634980 22223240

[126] Pérez de la Ossa N Carrera D Gorchs M Querol M Millán M Gomis M. Design and validation of a prehospital stroke scale to predict large arterial occlusion: the rapid arterial occlusion evaluation scale. Stroke. (2014) 45:87–91. 10.1161/STROKEAHA.113.003071 24281224

[127] Katz BS McMullan JT Sucharew H Adeoye O Broderick JP. Design and validation of a prehospital scale to predict stroke severity: Cincinnati Prehospital Stroke Severity Scale. Stroke. (2015) 46:1508–12. 10.1161/STROKEAHA.115.008804 25899242 PMC4442042

[128] Hastrup S Damgaard D Johnsen SP Andersen G Prehospital acute stroke severity scale to predict large artery occlusion: design and comparison with other scales. Stroke. (2016) 47:1772–6. 10.1161/STROKEAHA.115.012482 27272487

[129] Turc G Maier B Naggara O Seners P Isabel C Tisserand M Clinical scales do not reliably identify acute ischemic stroke patients with large-artery occlusion. Stroke. (2016) 47:1466–72. 10.1161/STROKEAHA.116.013144 27125526

[130] Bouckaert M Lemmens R Thijs V. Reducing prehospital delay in acute stroke. Nat Rev Neurol. (2009) 5:477–83. 10.1038/nrneurol.2009.116 19668246

[131] Caceres JA Adil MM Jadhav V Chaudhry SA Pawar S Rodriguez GJ. Diagnosis of stroke by emergency medical dispatchers and its impact on the prehospital care of patients. J Stroke Cerebrovasc Dis. (2013) 22:e610–4. 10.1016/j.jstrokecerebrovasdis.2013.07.039 24075587

[132] Buck BH Starkman S Eckstein M Kidwell CS Haines J Huang R. Dispatcher recognition of stroke using the National Academy Medical Priority Dispatch System. Stroke. (2009) 40:2027–30. 10.1161/STROKEAHA.108.545574 19390065 PMC2711028

[133] Jones SP Carter B Ford GA Gibson JM Leathley MJ McAdam JJ. The identification of acute stroke: an analysis of emergency calls. Int J Stroke. (2013) 8:408–12. 10.1111/j.1747-4949.2011.00749.x 22335960

[134] Kim SK Lee SY Bae HJ Lee YS Kim SY Kang MJ. Pre-hospital notification reduced the door-to-needle time for iv t-PA in acute ischaemic stroke. Eur J Neurol. (2009) 16:1331–5. 10.1111/j.1468-1331.2009.02762.x 19832903

[135] Yu RF San Jose MC Manzanilla BM Oris MY Gan R. Sources and reasons for delays in the care of acute stroke patients. J Neurol Sci. (2002) 199:49–54. 10.1016/S0022-510X(02)00103-X 12084442

[136] Brainin M Teuschl Y Kalra L. Acute treatment and long-term management of stroke in developing countries. Lancet Neurol. (2007) 6:553–61. 10.1016/S1474-4422(07)70005-4 17509490

